# Effective School Actions for Mitigating Seasonal Influenza Outbreaks in Niigata, Japan

**DOI:** 10.1371/journal.pone.0074716

**Published:** 2013-09-10

**Authors:** Koshu Sugisaki, Nao Seki, Naohito Tanabe, Reiko Saito, Asami Sasaki, Satoshi Sasaki, Hiroshi Suzuki

**Affiliations:** 1 Department of Health and Sports, Niigata University of Health and Welfare, Niigata, Japan; 2 School of Health Science, Faculty of Medicine, Niigata University, Niigata, Japan; 3 Department of Health and Nutrition, Faculty of Human Life Studies, University of Niigata Prefecture, Niigata, Japan; 4 Division of International Health, Niigata University Graduate School of Medical and Dental Sciences, Niigata, Japan; 5 School for Excellence in Educational Development, Soka University, Hachioji, Japan; 6 Department of Nursing, Niigata Seiryo University, Niigata, Japan; Harvard School of Public Health, United States of America

## Abstract

**Background:**

Japan has implemented various school actions during seasonal influenza outbreaks since the 1950's under the School Health Law. However, the effective duration, extent, and timing of closures remain unresolved.

**Materials and Methods:**

We conducted a retrospective study on the relationship between elementary class closures and influenza outbreak control during four consecutive influenza seasons from the 2004-2005 to 2007-2008 school years in Joetsu, Niigata, Japan. Among a total of 1,061 classes of 72 schools, 624 cases of influenza outbreaks were documented among 61 schools.

**Results:**

Class closures were carried out in a total of 62 cases in response to influenza outbreak, which was defined as a student absentee rate of greater than 10% due to influenza or influenza-like illness. Of these cases, two-day class closures were conducted the day after reaching a 10% student absentee rate in 28 cases and other types of closures were initiated in 34 cases. A markedly higher number of outbreak cases ended within one week for two-day class closures compared to the other types of closures (82.1% vs. 20.6%, respectively). The significant association between two-day class closures and interruption of an outbreak within one week was confirmed using a multivariable model adjusted for the season, grade, day of the week of an outbreak start, and absentee rate on the day of an outbreak start (OR, 3.18; 95% CI, 1.12–9.07; p = 0.030).

**Conclusions:**

Our results suggest that a two-day class closure carried out the day after reaching a 10% absentee rate is an effective approach for mitigating influenza outbreaks in elementary schools.

## Introduction

School-age children have the highest rates of infection by influenza virus and play a central role in facilitating its transmission within schools and the wider community during seasonal and pandemic outbreaks [1], [2]. School closure is one strategy for reducing the impact of H1N1 influenza pandemics [3], and consists of two types of measures [4]. The first is a proactive measure aimed at using school closures to reduce viral transmission within schools and subsequent spread of the virus into the wider community. The second is a reactive measure to high levels of absenteeism among students and staff, and involves the suspension of either the entire school or individual classes to limit viral spread. However, for both types of actions, the duration and extent of the school closure can have a large economic impact if parents of schoolchildren are required to stay home for caretaking [4], [5]. Thus, a better understanding of the measures that effectively limit the spread of influenza virus with minimal impact on normal school operation is needed.

Historically, Japan has adopted a unique system for school closure during seasonal influenza outbreaks that has been implemented since the 1950’s under the School Health Law. A variety of options for reducing influenza infections within schools are available: the closure of entire schools, specific grade levels, or individual classes; a later start of the school day; and cancellation of school activities in the afternoon. Analyses of influenza outbreak records dating back > 50 years among Japanese schoolchildren have suggested that school closures of less than 3 days are not effective for controlling the spread of infection, and that longer-duration school closure (> 5 days) markedly reduces the occurrence of secondary outbreaks [6]. We previously demonstrated the predictive value of a simple and practical detection method for triggering school closures soon after an influenza outbreak [7]. The analysis suggested that threshold influenza-related absentee rates of 5%, >4%, and >3% for one, two, or three consecutive days, respectively, are optimal for alerting school administrators to consider school closure. However, the proper duration, size, and timing of class closures have not been fully resolved.

Here, we conducted a retrospective study to assess the relationship between school actions and the control of influenza outbreaks in elementary schools during four consecutive influenza seasons using absenteeism data for school children infected with influenza and the class closure condition.

## Materials and Methods

### Study Location and Design

This study was conducted in Joetsu City, Niigata Prefecture, which is located in the Northeastern part of Japan and has an area of 973.32 km^2^ and a population of 208,626 as of 2008. We obtained data collected by the Joetsu City Board of Education during four influenza seasons from the 2004–2005 to 2007–2008 school years. During influenza season, each elementary classroom teacher assessed children as being infected with influenza virus based on oral reports of fever, coughing, sore throat, coryza, or direct reports from households, as required by the Niigata Prefecture governmental regulations. Almost all children with influenza were diagnosed by school doctors or local hospitals using the rapid antigen detection test and were treated with antiviral drugs. Public policy dictates that children with influenza cannot attend school until two days after the alleviation of fever and require written permission by a physician to return to school.

In Niigata Prefecture, school actions are recommended once daily student absentee rates reach greater than 10% due to influenza or influenza-like illness and are implemented by school principals based on the advice of school doctors and the Board of Education [8]. School actions vary and include closures of the entire school, specific grade levels, or individual classes, a later start of the school day, and cancellation of school activities in the afternoon. During influenza season in Joetsu City, all elementary schools submit a daily report (excluding public holidays and weekends) of total absenteeism due to influenza or influenza-like illness and the number of students with influenza in each class and the type of school actions undertaken after an influenza outbreak to the city Board of Education.

An outbreak case was defined as a student absentee rate of greater than 10% due to influenza or influenza-like illness. Instances in which the daily absentee rate of a class was less than 10% during the week following an outbreak were defined as an interruption of the outbreak. Based on these definitions, it was judged whether an outbreak ended within one week. The number of days from the first day of greater than 10% absentee rates to the last day of a 10% absentee rate was defined as the outbreak duration. Saturday, Sunday, and national holidays were included in the outbreak duration. Cases in which an outbreak started on a Friday were excluded from this study because specific school actions were not taken. A two-day class closure and a typical weekend of Saturday-Sunday were not considered to be equal concerning school actions as teachers may have given students health instructions, such as limiting time in public spaces, at the time of class closure [9].

### Types of Class Closure

**Figure 1:**
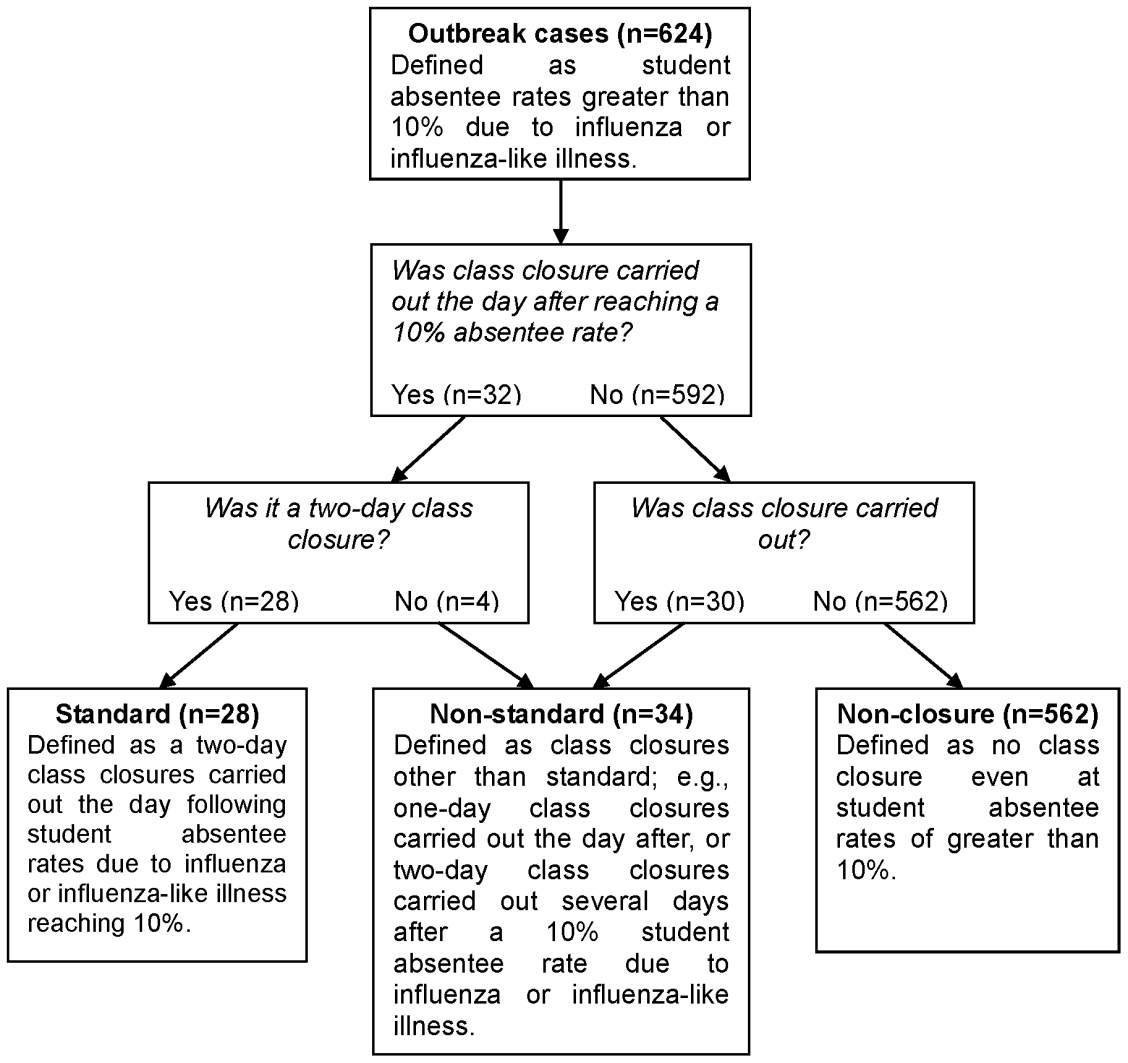
Types of Class Closure.

Class closure was classified as three distinct types: Standard, Non-standard, and Non-closure (Figure 1). A standard class closure was defined as a two-day class closure carried out the day following student absentee rates due to influenza or influenza-like illness reaching 10% (Standard). Class closures other than standard class closures were defined as a non-standard class closure (Non-standard). Examples of Non-standard closures include one-day class closures carried out after a 10% student absentee rate (One-day) or two-day class closures carried out two days or more after a 10% student absentee rate (Delayed) due to influenza or influenza-like illness. No class closure, even at student absentee rates of greater than 10%, was defined as a non-closure (Non-closure). Finally, Non-standard + Non-closure was defined as Combined.

### Statistical Analysis

The effect of class closures as a reactive measure against influenza outbreak in schools was assessed using two outcome measurements: outbreak duration and interruption of an outbreak within one week. To analyze associations between the type of class closure and the outbreak duration, a linear regression model was used for calculating the difference in the number of outbreak days (Δday) between Standard and Non-standard. The interruption of an outbreak within one week represented the primary outcome for the effect of Standard class closure and was assumed to be a dependent variable. Independent variables consisted of the types of class closure (Standard vs. Non-standard + Non-closure). A logistic regression model was used for calculating the unadjusted and multivariable adjusted odds ratio (OR) and parameter estimates for the effect of Standard class closures. Unadjusted and multivariable adjusted OR and 95% confidence intervals (CI) were also calculated. In the multivariable models, four potential confounding factors were adjusted: season (2004–2005, 2005–2006, 2006–2007, and 2007–2008), grade (from 1–6), absentee rate on the start day of an outbreak, and day of the week of an outbreak start (Monday, Tuesday, Wednesday, or Thursday). The identical analysis was performed using data for outbreaks that began on a Monday, as 92.9% outbreaks associated with cases of Standard began on this day. For data of Monday outbreaks only, three factors exclusive of day of the week for starting an outbreak were adjusted. All statistical analyses were performed using IBM SPSS Statistics Desktop Version 19.0 for Windows. Statistical significance was defined as p < 0.05.

## Results

Data for the total absenteeism due to influenza or influenza-like illness in each class and the type of school action for 54 elementary schools with 537–559 classes from the first to sixth grades (6–11 year-old students) were obtained from the Joetsu City Board of Education from the 2004–2005 to 2007–2008 seasons. After excluding small schools with less than two classes per grade, 1,061 classes (median number of children, 29; range, 17–42) from 72 schools were analyzed during four consecutive years (Table 1). Finally, 624 cases from a total of 61 schools experienced influenza outbreaks. A total of 62 class closures were carried out, and almost half of the closures were implemented during the 2004–2005 season.

**Table 1:** Number of classes, outbreak cases, and class closures examined in this study.

School year	2004–2005	2005–2006	2006–2007	2007–2008	Four-year total
No. of classes	266	267	265	263	1061
No. of children in each class, median (min-max)	29 (17–39)	29 (17–42)	29 (16–40)	29 (16–40)	29 (17–42)
No. of outbreak schools	18	13	14	16	61
No. of outbreak cases	198	128	156	142	624
No. of class closures	32	2	18	10	62
Class closure duration, median (min-max)	2 (1–3)	1 (1–1)	2 (1–2)	2 (2–2)	2 (1–3)

Influenza outbreaks occurred in 567 individual classes during four influenza seasons. More than one outbreak occurred in 25 of 172 (14.6%) classes during the 2004–2005 season, 9 of 117 (7.7%) classes during the 2005–2006 season, 13 of 143 (9.1%) classes during the 2006–2007 season, and 7 of 135 (5.2%) classes during the 2007–2008 season. Three outbreaks occurred in one class during the 2004–2005 season and two classes during the 2005–2006 season. The total number of outbreak cases was 624, but almost all influenza outbreaks in a school occurred at the unit of individual classes. Entire school closures were not reported, even during the high-volume influenza season of 2004–2005, during the four consecutive influenza seasons.

Among the 624 outbreak cases, 28 cases were Standard, 34 cases were Non-standard, and the remaining cases were classified as Non-closure despite the recommendation of action being taken at 10% absentee rates (Table 2). The Standard group exhibited the shortest outbreak duration (6.0±2.7 days) compared with Non-standard or Non-closure groups, and had the highest rate of an outbreak being interrupted within one week (82.1%). The absentee rate was the highest on Mondays, with 51.9% of outbreaks starting on this day. The peak of an outbreak typically occurred on Monday. Standard closures were started on either Tuesday (92.9%) or Wednesday (7.1%).

**Table 2:** Characteristics of influenza outbreaks and class closures.

Parameter	Total	Standard*	Non-standard†	One-day‡	Delayed§	Non-closure∥	Combined¶
***No. of outbreak cases (%)***	***624 (100.0%)***	***28 (100.0%)***	***34 (100.0%)***	***4 (100.0%)***	***30 (100.0%)***	***562 (100.0%)***	***596 (100.0%)***
Outbreak duration, days, mean (± S.D)	7.0 (±5.2)	6.0 (±2.7)	10.9 (±5.4)	3.3 (±1.5)	12.0 (±4.9)	6.8 (±5.1)	7.1 (±5.2)
No. of cases with interruption of outbreak within one week (%)	320 (51.3%)	23 (82.1%)	7 (20.6%)	4 (100.0%)	3 (10.0%)	290 (51.6%)	297 (49.8%)
Absentee rate at start day, %, mean(± S.D)	16.2 (±7.0)	29.4 (±11.5)	16.4 (±5.9)	21.2 (±6.2)	15.7 (±5.7)	15.6 (±6.1)	15.6 (±6.1)
***Day of the week of outbreak start (%)***							
Monday	324 (51.9%)	26 (92.9%)	13 (38.2%)	1 (25.0%)	12 (40.0%)	283 (50.4%)	296 (49.7%)
Tuesday	108 (17.3%)	2 (7.1%)	9 (26.5%)	1 (25.0%)	8 (26.7%)	99 (17.6%)	108 (18.1%)
Wednesday	97 (15.5%)	0 (0.0%)	8 (23.5%)	2 (50.0%)	6 (20.0%)	89 (15.8%)	97 (16.3%)
Thursday	95 (15.2%)	0 (0.0%)	4 (11.8%)	0 (0.0%)	4 (13.3%)	91 (16.2%)	95 (15.9%)
Friday	-----	-----	-----	-----	-----	-----	-----
Day of the week of peak absentee rate (%)							
Monday	237 (38.0%)	17 (60.7%)	14 (41.2%)	1 (25.0%)	13 (43.3%)	206 (36.7%)	220 (36.9%)
Tuesday	140 (22.4%)	0 (0.0%)	8 (23.5%)	1 (25.0%)	7 (23.3%)	132 (23.5%)	140 (23.5%)
Wednesday	93 (14.9%)	0 (0.0%)	9 (26.5%)	2 (50.0%)	7 (23.3%)	84 (14.9%)	93 (15.6%)
Thursday	88 (14.1%)	10 (35.7%)	3 (8.8%)	0 (0.0%)	3 (10.0%)	75 (13.3%)	78 (13.1%)
Friday	66 (10.6%)	1 (3.6%)	0 (0.0%)	0 (0.0%)	0 (0.0%)	65 (11.6%)	65 (10.9%)
***Day of the week for starting class closure (%)***	***N = 62***	***N = 28***	***N = 34***	***N = 4***	***N = 30***		
Monday	0 (0.0%)	0 (0.0%)	0 (0.0%)	0 (0.0%)	0 (0.0%)	-----	-----
Tuesday	38 (61.3%)	26 (92.9%)	12 (35.3%)	1 (25.0%)	9 (30.0%)	-----	-----
Wednesday	7 (11.3%)	2 (7.1%)	5 (14.7%)	1 (25.0%)	4 (13.3%)	-----	-----
Thursday	10 (16.1%)	0 (0.0%)	10 (29.4%)	2 (50.0%)	10 (33.3%)	-----	-----
Friday	7 (11.3%)	0 (0.0%)	7 (20.6%)	0 (0.0%)	7 (23.3%)	-----	-----

* Standard: two-day class closure carried out the day after reaching or exceeding a 10% student absentee rate due to influenza.

† Non-standard: closures other than Standard.

‡ One-day: one-day class closure carried out after a 10% student absentee rate.

§ Delayed: two-day or three-day class closures carried out two days or more after a 10% student absentee rate.

∥ Non-closure: no class closure even at 10% absentee rates.

¶ Combined: non-standard + non-closure.

The 34 Non-standard closure cases were further subdivided into One-day (n = 4) and Delayed closures (n = 30). All cases of One-day interrupted the outbreak within one week (100%) and outbreak duration (3.3±1.5 days) was the markedly shorter than the case of Delayed closure, which was the most ineffective for both outbreak interruption (10.0%) and outbreak duration (12.0±4.9 days).

Single-variable analysis detected a significant association between Standard class closures and the interruption of an outbreak within one week (OR, 4.63; 95% CI, 1.74–12.34; p = 0.002) (Table 3). This association was also demonstrated using the multivariable model adjusted for the season, grade, absentee rate on the start day of outbreak, and day of the week for starting an outbreak (OR, 3.18; 95% CI, 1.12–9.07; p = 0.030). The outbreak duration for Standard class closures was –4.98 days shorter than Non-standard (p<0.001) from the single-variable analyses, and –4.09 days shorter than Non-standard (p = 0.008) from the multi-variable analyses. For the analysis of Standard closures, particularly for outbreaks starting on Monday, the OR was relatively high in both the single- (OR, 3.16; 95% CI, 1.16–8.60; p = 0.025) and multi-variable (OR, 3.10; 95% CI, 1.10–9.07; p = 0.039) analyses (Table 4).

**Table 3:** Effect of Standard class closure on outbreak duration.

Outbreak duration	Δday*	(95% CI)	P value
Unadjusted	–4.98	(–7.22, –2.74)	< 0.001
Adjusted‡	–4.09	(–7.08,–1.10)	0.008
Interruption within one week	OR†	(95% CI)	P value
Unadjusted	4.63	(1.74, 12.34)	0.002
Adjusted‡	3.18	(1.12, 9.07)	0.030

* Difference in the number of outbreak days compared with non-standard class closures.

† Combined (non-standard + non-closure) was used as a reference.

‡ Adjusted for the season, grade, absentee rate at start day of outbreak, and day of the week for starting an outbreak.

**Table 4:** Effect of Standard class closure (Monday outbreaks only) on outbreak duration.

Outbreak duration	Δday*	(95% CI)	P value
Unadjusted	–5.23	(–7.88, –2.58)	< 0.001
Adjusted‡	–4.50	(–7.53, –1.47)	0.005
Interruption within one week	OR†	(95% CI)	P value
Unadjusted	3.16	(1.16, 8.60)	0.025
Adjusted‡	3.10	(1.10, 9.07)	0.039

* Difference in the number of outbreak days compared standard with non-standard class closures.

† Combined (non-standard + non-closure) were used as a reference.

‡ Adjusted for the season, grade, and absentee rate at start day of outbreak.

## Discussion

In this study, we attempted to clarify the relationship between school actions and the control of influenza outbreaks during four consecutive influenza seasons (2003–2007) in elementary schools in Joetsu City, Niigata, Japan. Our analyses lead us to conclude that during an influenza outbreak in a class, a two-day class closure carried out the day after the student absentee rate reaches 10% or greater is effective for mitigating outbreaks in elementary schools.

Several reports have suggested that school closures may be effective for limiting the severity of outbreaks during influenza season [9]–[13]. For example, an Israeli study showed a significant reduction in respiratory infections during school closures that were initiated by a teacher strike [9]. A model study in France also estimated that school holidays prevented 16%-18% of seasonal influenza cases and led to a 20%–29% reduction of influenza transmission among school children [10]. Furthermore, several studies have reported the effectiveness of non-pharmaceutical approaches, such as class dismissal, and school, class, reactive, and proactive closures, for reducing the spread of influenza virus during seasonal or pandemic influenza [13]–[20]. In contrast, school closure had no effect for community transmission or reduction of absenteeism due to influenza or influenza-like illness [21], [22]. However, our present results contradict the findings of these previous reports. The apparent discrepancy between these studies may be due to differences in the type of action taken in response to an outbreak, namely entire school closure or class closure, and the examination of data from only a single season or four continuous seasons, as was performed in the present study.

The World Health Organization has proposed that two types of school closure, involving either proactive or reactive measures, be undertaken to control influenza pandemics; however, the efficacy of each approach for seasonal influenza remains a source of debate [23]. Here, we found that a reactive approach involving two-day class closures led to the shortest duration of seasonal influenza outbreaks and was more likely to interrupt the outbreak within one week.

We presumed that influenza outbreaks at schools occur on the scale of an entire grade or school. However, our study of four consecutive influenza seasons found that entire school closures were not reported, even during a high-volume influenza season, and that nearly all influenza outbreaks in a single school occurred at the level of individual classes. These findings suggest that school actions should be conducted at the class level as a basic strategy. However, as our study analyzed a relatively small number of schools during only four influenza seasons, further studies are warranted to confirm these findings.

During four influenza seasons, more than one outbreak occurred in 9.5% of classes during each season, although three outbreaks occurred in only one class during the 2004–2005 season and in two classes during the 2005–2006 season. These variations likely occurred due to ineffective school actions or as a result of infection with different viral strains.

The timing of a class closure is crucial to control an influenza outbreak. In Niigata Prefecture, school principals can implement school actions when the absentee rate of a class is more than 10% of the students in a class. We have previously reported that many classes with absentee rates of more than 10% had increased numbers of infections and likely contributed to the expansion of the influenza outbreak to other classes [24]. However, our present analyses revealed that when two-day class closures were implemented upon absentee rates reaching 10% (Standard class closure), outbreaks were effectively interrupted within one week. Based on these findings, we conclude that class closures should be implemented once student absentee rates reach 10% in a class. We previously demonstrated the predictive value of a simple and practical detection method for triggering school closures in response to influenza outbreaks [7]. Specifically, threshold influenza-related absentee rates of 5%, >4%, or >3% for one, two, or three consecutive days, respectively, were optimal for initiating a school closure [7]. In this study, although 10% absentee rates were used, further investigations to determine the most effective criteria for implementing school actions are warranted.

As our study mainly focused on seasonal influenza outbreaks, the appropriate duration of school closure likely differs from that needed for controlling pandemics. Japan has implemented school actions during seasonal influenza outbreaks since the 1950’s under the School Health Law. Presently, the main purpose of class closure is the interruption of an influenza outbreak within the school and not the control of an influenza outbreak in the community. However, as a common family in Japan consists of two working parents and 1-2 children, the duration of a school closure is also quite important in terms of childcare and the economy. For instance, in Australia, 45% of parents of asymptomatic students reported taking at least one day off work to care for their child [25]. In the USA, 16%–22% of households reported that a family member had missed work and lost pay to care for infected schoolchildren [26], [27]. Therefore, minimal, but effective, school actions would help limit lost work days for the parents of uninfected children, thereby lessening the impact of school closures initiated in response to influenza infection. Our findings suggest that two-day class closure is an effective strategy for interrupting influenza outbreaks in a class. Furthermore, as the absentee rates during an outbreak were highest on Monday, class closures were typically initiated on Tuesday or Wednesday. Thus, these findings support the view that two-day class closures, such as Tuesday-Wednesday, should be undertaken immediately once class absentee rates reach 10%.

In the present study, as a unique case, a class closure was implemented on a Friday. Although this was equivalent to a three-day class closure, it was ineffective and failed to interrupt the outbreak. Furthermore, as many students visit public areas and participate in sports and educational activities during the weekend, the potential for virus transmission among schoolchildren and the public increases. Such activities on the weekend likely resulted in the highest absentee rates on Mondays, because the incubation period of influenza is approximately 1–2 days. These results suggest that information on behavior and the lifestyle of children and their families during weekends or holidays is important for developing effective strategies against influenza outbreaks in schools and communities.

Although Non-standard closures were shown to be relatively ineffective at mitigating an influenza outbreak within a class, subgroup analyses revealed that one-day class closure effectively interrupted outbreaks within one week and resulted in outbreaks of shorter duration than those controlled by Standard closures. However, as only 4 cases of one-day closure were reported, more cases are required to reach a statistically significant conclusion. In our previous study, less than 2% of principals responded one-day class closures were needed [8]. Thus, it may be difficult to evaluate the efficacy of this class-closure response. Two important factors for deciding school actions during influenza season are the quality of diagnosis of influenza infections and vaccination rate of school children. Currently, almost all students with suspected infections are diagnosed by rapid test kits in clinics and hospitals in Japan. Although the effectiveness of vaccination is related to the match between the immunizing antigen and epidemic virus, programs for increasing vaccination rates in conjunction with reactive school closures are expected to limit influenza outbreaks [28]. In addition, universal influenza vaccination for school children was effective in reducing the number of class cancellation days and absenteeism rates [29].

Several limitations of our study warrant mention. Firstly, as all data were collected by individual schools, it is possible that we had incomplete data on influenza cases and rates of absenteeism. Secondly, as we analyzed only larger schools with more than two classes in each grade, it is possible that trends in smaller schools would show different outcomes. Thirdly, the effects of class closures on inter-class transmission were not considered in the statistical models. This is a limitation of the statistical analysis and should be considered when interpreting the present results. Finally, the actual vaccination rate of students in Joetsu City was not known.

In conclusion, our retrospective analysis of school actions in response to influenza-related absenteeism during four consecutive influenza seasons suggests that two-day class closure once class absentee rates reach 10% is an effective action for interrupting influenza outbreaks in elementary schools.
